# Use of Oral Prednisolone and a 3-Phase Bone Scintigraphy in Patients with Complex Regional Pain Syndrome Type I

**DOI:** 10.3390/healthcare8010016

**Published:** 2020-01-09

**Authors:** Seunghun Park, Hyun-Jun Kim, Dong Kyu Kim, Tae Hee Kim

**Affiliations:** 1 Department of Rehabilitation Medicine, Konkuk University Chungju Hospital, Chungju 27478, Korea; silveryluna@gmail.com (S.P.); peyous@hanmail.net (D.K.K.); 2 Department of Obstetrics & Gynecology, School of Medicine, Konkuk University, Chungju 27478, Korea; icarus@kku.ac.kr; 3 Research Institute of Medical Science, Konkuk University School of Medicine, Seoul 05029, Korea

**Keywords:** complex regional pain syndromes, steroids, radionuclide imaging, high dose, low dose

## Abstract

To compare the treatment effects of a high-dose and low-dose oral steroid regimen based on changes in the radioisotope uptake ratio (RUR) observed from three-phase bone scintigraphy (TPBS) in patients with complex regional pain syndrome type I (CRPS I), we retrospectively analyzed data of 34 patients with CRPS I from traumatic brain injury and stroke. Depending on the dose of steroid administered, patients were divided into high-dose (*n* = 14) and low-dose steroid groups (*n* = 20). We compared the severity scores, Kozin’s classification scores, and RUR observed from TPBS between the two groups. There were significant changes in the severity scores and Kozin’s classification between the baseline and 2 weeks from baseline (*p* < 0.05), however, there were no significant differences in terms of changes in the scores, classification, or the RUR observed from TPBS at 2 weeks from baseline (*p* > 0.05). There were no treatment-emergent adverse events (TEAEs) such as blood pressure elevation, impaired glycemic control, or gastrointestinal disturbances. Our results indicate that the efficacy profile of a low-dose oral steroid regimen is comparable to that of a high-dose regimen in alleviating symptoms in CRPS I patients. However, additional prospective, large-scale, multi-center studies are warranted to confirm our results.

## 1. Introduction

Complex regional pain syndrome (CRPS) is a chronic neurological disorder that commonly affects the upper extremities, lower extremities, hands, or feet, with pain often spreading to the entire limbs [1]. CRPS can be divided into two types: type I (CRPS I), accounting for 90% of the total cases of CRPS, and type II (CRPS II). Typical causes of CRPS I include minor traumas, fractures, and complications after stroke. Especially in the case of CRPS that occurred after a stroke, in some studies, the incidence was as high as 50%. CRPS II is characterized by the definite presence of nerve injury [2].

Despite recent advancements in understanding CRPS I, it still poses a diagnostic and therapeutic dilemma for clinicians. Its pathophysiology, clinical course, and optimal treatment modalities remain uncertain [3]. Patients with CRPS I are vulnerable to severe disability and intractable pain; they are burdened with high healthcare costs unless appropriately diagnosed and treated [4].

Previously, CRPS I was typically diagnosed based on clinical symptoms and signs without an objective diagnostic modality [5]. The International Association for the Study of Pain (IASP) coded the name and diagnostic criteria for CRPS I in 1994. Since that time, considerable efforts have been made to establish accurate, valid diagnostic criteria for CRPS I [6], and diagnostic consensus criteria were established at an expert meeting held in Budapest in 2003 [7]. According to these diagnostic criteria, a diagnosis of CRPS I can be made if a patient has at least one symptom in all four symptom categories and at least one sign in two or more sign categories [8]. In addition to clinical findings, several additional diagnostic modalities have been used to establish a diagnosis of CRPS 1, including three-phase bone scintigraphy (TPBS). Although TPBS-associated diagnosis of CRPS has been considered controversial, several studies have recently reported on the relationship between the two. Furthermore, TPBS is considered an effective diagnostic modality for CRPS I [9]. It has been reported that quantitative analyses of blood-pool phase and delayed-phase images of the hand based on a TPBS are helpful in making a diagnosis of CRPS I in patients presenting with definite signs and symptoms [10,11,12]. TPBS is no longer used to purely diagnose CRPS, although evidence suggests that TPBS may be an effective modality to determine treatment response in patients with an established diagnosis of CRPS I [13,14].

To date, various modalities have been used to treat CRPS I; these include non-steroidal anti-inflammatory drugs (NSAIDs), anticonvulsants, muscle relaxants, intrathecal baclofen (ITB) therapy, stellate ganglion block, nerve block, spinal cord stimulation, and percutaneous electric nerve stimulation (PENS) [15,16]. Among these, steroids are considered more effective with a relatively shorter treatment duration [17].

Currently, an oral steroid regimen is the most frequently prescribed treatment for CRPS I. However, this treatment remains problematic because it produces adverse effects such as hyperglycemia, hypertension, peptic ulcers, and adrenal dysfunction. Therefore, there is a need to be more careful when prescribing steroids in stroke patients with many comorbid diseases. This poses a safety issue in patients [17,18].

Given this background, we conducted this study to compare the treatment effects of a high-dose oral steroid regimen to the effects of a low-dose regimen based on changes in the radioisotope uptake ratio (RUR) observed from TPBS scans in patients with CRPS I.

## 2. Materials and Methods

### 2.1. Study Patients and Setting

This single-center, retrospective study was conducted in patients with CRPS I who were hospitalized at the department of physical medicine and rehabilitation within our medical institution (Konkuk University Chungju Hospital, Chungju, Korea) between October 2012 and December 2016.

We included patients who met the diagnostic criteria for CRPS I, proposed by the IASP (International Association for the Study of Pain) in 1994, in the hemiplegic upper limbs [7]. Included patients did not have contraindications for steroid therapy or other notable concurrent diseases that might cause CRPS I, and had no history of trauma or peripheral nerve damages at the affected sites. Also, included patients were not currently on any medications that might have interacted with steroid therapy during treatment period. We also excluded patients who received local steroid injections between October 2012 and December 2016, those who were administered oral steroids for other diseases, and those who were deemed to be ineligible for study participation according to our judgment.

Therefore, in this study, we retrospectively analyzed the data of 34 patients (*n* = 34), including their RURs obtained from TPBS images and the treatment dose. This study was conducted in accordance with the Declaration of Helsinki, and the protocol was approved by the Institutional Review Board (IRB) of our medical institution (IRB approval # KUCH 2019-04-014). The requirement for informed consent was waived owing to the retrospective design of the study. 

### 2.2. TPBS Protocol

**Figure 1:**
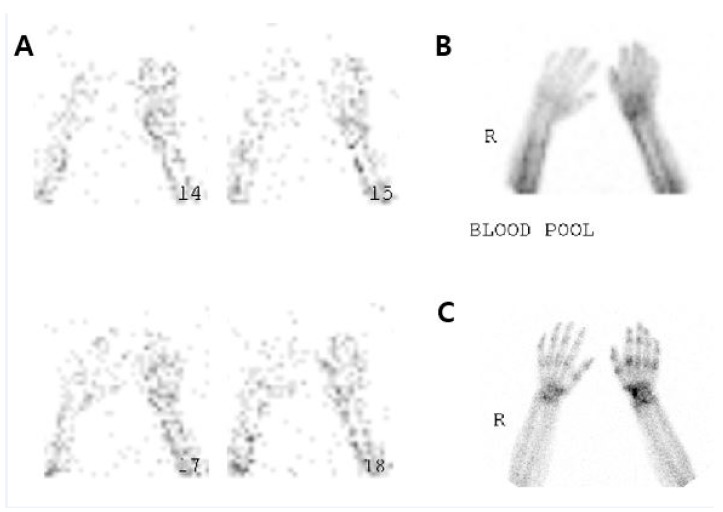
The three-phase bone scintigraphy images.

Each patient underwent two TPBS scans using a dual-head gamma camera (FORTE; Philips Medical Systems, Cleveland, OH, USA) mounted to a low-energy, high-resolution collimator: one prior to the treatment with steroids, and one within 5 days after treatment. Images were acquired in three phases. During the first phase, perfusion-phase images were obtained immediately after the injection of 1110 MBq of technetium-99 m methylene diphosphonate in the lower extremities. During the second phase, blood pool phase images were obtained five minutes after the injection of radioisotope. During the third phase, delayed-phase images were obtained 2–4 h after the injection of radionuclide [19]. We fixed the location of the patient’s hand in all TPBS images using a fixation device, extending both the wrists and phalangeal joints in a prone position. After obtaining the TPBS images before and after the treatment, we calculated the RURs for each patient’s hands and compared the affected and unaffected side (Figure 1). 

After fixing the location of the hand for all of the three-phase bone scintigraphy (TPBS) images by using a fixation device, we extended both the wrist and phalangeal joints when placing both wrists in a prone position. Then, we calculated the radioisotope uptake ratio (RUR) based on the TPBS and compared it between the affected and unaffected side, as suggested by previous research [20].

### 2.3. Treatment Protocol

**Figure 2:**
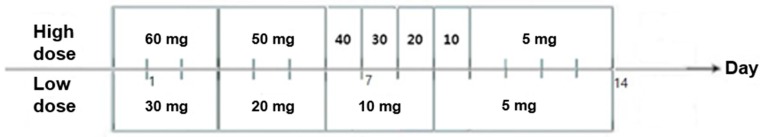
Protocol for oral corticosteroid therapy.

Patients received oral prednisolone (Solondo Tab^®^; YuHan Corp., Seoul, Korea) for two weeks. Depending on the dose received, patients were divided into two groups: the high-dose group (total dose: 450 mg) and the low-dose group (total dose: 200 mg). The patients in the high-dose group received oral prednisolone, 60 mg per day, initially, with a rapid decrease over two weeks. The daily dose was set at 60 mg for the first 3 days, then 50 mg for the next 3 days, and the dose was then decreased by 10 mg per day over a 4-day interval. From day 11 to day 14, the daily dosage was 5 mg per day. Following this, we discontinued the steroid therapy. For low dose prednisolone dosing, we arbitrarily set its initial dose at 30 mg. Thus, the patients received oral prednisolone at an initial dose of 30 mg daily for 3 days, a dose of 20 mg over the next 3-day interval, and a dose of 10 mg for the subsequent 3-day interval. From day 10 to day 14, the patients in the low-dose group received a 5-day course of 5 mg prednisolone, after which we discontinued the steroid therapy in the low-dose group (Figure 2).

Both groups of patients also received the joint movement and infrared therapy.

### 2.4. Patient Evaluation Criteria

Baseline and clinical characteristics of the patients included age, sex, disease duration, affected side, and causes of disease. These were evaluated through a retrospective review of medical records.

In both groups, outcomes at two weeks from baseline were evaluated based on severity scores (Appendix A) suggested by Sung et al. reflecting the degree of hypersensitivity, pain, and swelling and Kozin’s classification scores (Appendix B) reflecting pain and edema arising from hypersensitivity of the hand and the rotational angle of the shoulder joint [21,22,23]. We also evaluated changes in the RUR based on TPBS scans at two weeks from baseline in both groups. Thus, severity scores, Kozin’s classification scores, and TPBS scans served as outcome measures in the current study.

For the efficacy assessment, we compared changes in the outcome measures at two weeks from baseline between the two groups. Based on a previously published study showing that scintigraphy is useful for predicting therapeutic response, and that the RUR based on TPBS scans has a significant correlation with the disease course in patients with CRPS I, we measured the RUR based on TPBS scans [24].

For safety assessment, we retrospectively evaluated the data, including treatment-emergent adverse events (TEAEs) (e.g., blood pressure elevation, impaired glycemic control, body weight gain, and gastrointestinal disturbances).

### 2.5. Statistical Analysis

All data were expressed as mean ± SD (SD: standard deviation). Statistical analysis was performed using SPSS 12.0 for Windows (SPSS Inc., Chicago, IL, USA). To identify the statistical differences between clinical characteristics, we performed an independent *t*-test and the Pearson’s chi-square test. In addition, we used the Wilcoxon signed-rank test to compare treatment outcomes at baseline and at two weeks in each group. A *p*-value of < 0.05 was considered statistically significant.

## 3. Results

### 3.1. Baseline Characteristics of the Patients

We performed a retrospective analysis using data from medical records for a total of 34 patients (14 men and 20 women, mean age = 63.4 ± 5.3 years). Depending on the dose of steroid received, we divided patients into two groups: the high-dose steroid group (*n* = 14) and low-dose group (*n* = 20). In our study, underlying conditions included 12 cases of ischemic cerebral infarction, 14 cases of hemorrhagic cerebral infarction, and 8 cases of traumatic brain injury (TBI). Baseline characteristics of the patients are shown in Table 1.

### 3.2. Efficacy Outcomes

**Figure 3:**
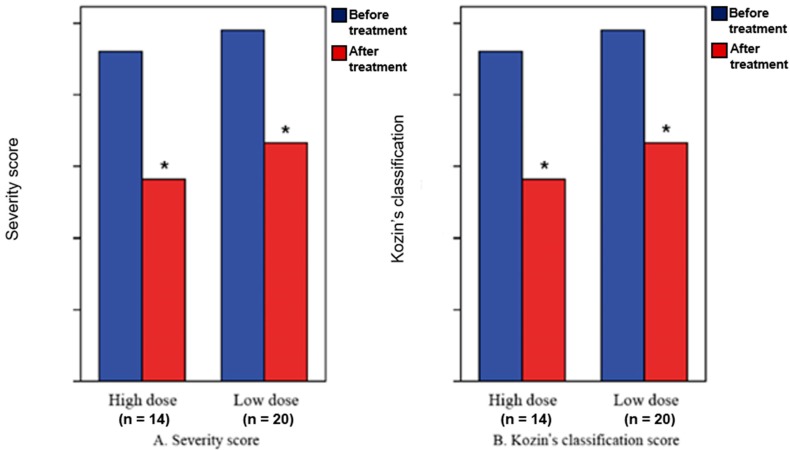
Severity scores (**A**) and Kozin’s classification scores (**B**). * Statistical significance at *p* < 0.05.

All 34 patients showed improvement in clinical symptoms; all exhibited significant changes in their severity scores and Kozin’s classification scores at two weeks from baseline (*p* < 0.05). However, the two treatment groups showed similar improvements between the two time-points (*p* > 0.05) (Table 2, Figure 3).

There were significant changes in the severity scores and Kozin’s classification scores at 2 weeks from baseline (*p* < 0.05), although there were no significant differences in the severity scores and Kozin’s classification scores at 2 weeks from baseline between the high-dose group and low-dose group (*p* > 0.05).

**Figure 4:**
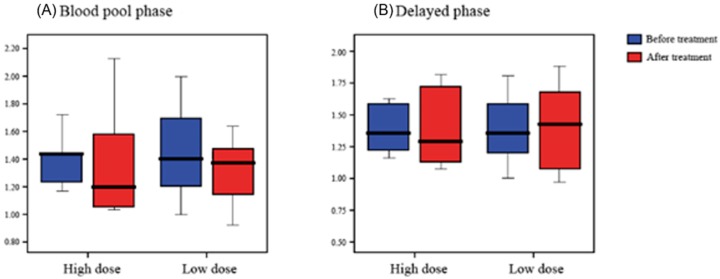
The radioisotope uptake ratio Blood pool phase (**A**) and Delayed phase (**B**) based on three-phase bone scintigraphy scans.

Comparing the RUR before and after treatment, in the blood pooling phase, the average ratio after treatment decreased in both groups, but in the delayed phase, it was observed that the average ratio increased in the low-dose group. We observed no significant difference of RUR in the TPBS between baseline and two weeks in the high-dose group and the low-dose group (*p* > 0.05) (Table 3, Figure 4).

In this study, the difference in TPBS scan was not significant when using high and low doses of steroid, but the patient’s severity score and Kozin’s classification score were statistically significant regardless of steroid dose.

### 3.3. Safety Outcomes

In this study, high- and low-dose steroid therapy was associated with the symptom of sour stomach in one patient from each of the two groups. The symptom demonstrated by the patient in each group was relatively mild and recovered rapidly after persisting for a day or two. Except for the two aforementioned patients, no other patients reported any side effects associated with steroid therapy, such as blood pressure elevation, impaired glycemic control, and weight gain, in either treatment group.

## 4. Discussion

The feasibility of anti-inflammatory therapies for the treatment of CRPS I has been studied in randomized controlled trials (RCTs) [25]. The advantages of these therapies include low costs and ease of administration. However, the efficacy of oral prednisolone in relieving pain has been reported to be better than oral piroxicam in patients with post-stroke acute CRPS [26,27]. Moreover, placebo-controlled RCTs have also shown that prednisolone or methylprednisolone were effective in improving symptoms in patients with early/acute CRPS and in those with post-stroke hemiplegia [28,29]. In addition, another RCT found that patients with post-stroke CRPS who were treated with prednisolone at a dose of 40 mg/day showed significantly more improvement over the course of a month than patients who were treated with piroxicam [27]. Furthermore, Braus et al. reported that 91% of patients with post-stroke CRPS achieved a significant improvement when treated with a 2-week course of methylprednisolone at four divided doses of 32 mg/day or a more than 2-week course of methylprednisolone at a tapered dose [29].

Despite its efficacy, administration of high-dose steroids in CRPS patients may yield side effects such as uncontrolled diabetes mellitus, hypertension, and gastrointestinal bleeding. Patients with brain injury and those with concurrent underlying diseases should be treated with special care when treated with steroids. Discontinuation or avoidance of high-dose steroids may be recommended for these patients because adverse effects may occur depending on steroid dose and duration [30]. Therefore, in these patients, a low-dose steroid regimen may be preferred. In this study, all patients with side effects showed symptoms of sour stomach despite the use of high-dose steroid therapy, possibly due to the steroid therapy being administered in a relatively short period. In addition, it has been shown that the treatment of low-dose therapy may be sufficiently effective compared to high-dose steroid therapy. Additionally, as we already know, low-dose therapy has fewer side effects. With these considerations in mind, we carefully suggest that low-dose steroid therapy may be a more effective treatment for CRPS in post stroke patients; however, further research is warranted to validate this. As mentioned earlier, TPBS is not used only as a diagnostic tool. Moreover, according to Dia et al. [13] and Emel et al. [14], TPBS can be used to evaluate the effect of treatment in CRPS patients. Our results showed that the difference in TPBS scans was not significant when using high and low doses of steroid, but the patient’s severity score and Kozin’s classification score were statistically significant regardless of the steroid dose. Our results showed that there was no significant difference in the treatment effects between the high-dose and low-dose groups. Thus, low-dose steroid therapy could be used instead of high-dose steroid therapy; however, this warrants further large-scale studies.

Our study had some limitations. First, we evaluated the condition of a small number of patients without classification by patient severity. Second, we only evaluated the data of patients who were hospitalized at a single medical institution. We could not therefore completely rule out the possibility of selection bias. Third, we evaluated patients after only a short period of post-treatment follow-up. Fourth, we failed to evaluate the range of the motion of joints and hyperalgesia constituting the symptoms of CRPS I. This is not only because our study’s patients were diagnosed with CRPS I after an average of 48.7 days following a stroke, but also because most of them were transferred to other medical institutions after a 2-week treatment period. Fifth, we failed to assess the long-term efficacy of steroid therapy. Sixth, we could not completely explain the effect of low-dose steroids because increased RUR was observed in delayed phase after treatment. Seventh, the retrospective study design may limit data collection, and patients with post-stroke pain syndrome exhibit symptoms similar to CRPS, which cannot be completely ruled out with the use of only one piece of diagnostic equipment. The simultaneous use of diagnostic tests (such as thermal image, quantitative sensory testing or quantitative sudomotor axon reflex test) for diagnosis would help distinguish these patients.

## 5. Conclusions

In conclusion, our results suggest that the effect of low-dose oral steroid regimen was equivalent to that of a high-dose regimen in alleviating symptoms in stroke patients with pain at the hemiplegic upper limbs. However, further prospective, large-scale, multi-center studies are warranted to confirm our results.

## Figures and Tables

**Table 1:** Baseline characteristics of the patients of each group.

Variables	Values	
	High-Dose Steroid Group (*n* = 14)	Low-Dose Steroid Group (*n* = 20)
Age (years)	62.4 ± 4.7	63.8 ± 5.6
Sex		
Men	6 (42.9%)	8 (40%)
Women	8 (57.1%)	12 (60%)
Side of involvement		
Right	6 (42.9%)	8 (40%)
Left	8 (57.1%)	12 (60%)
Time from onset (days)	50.3 ± 5.8	49.7 ± 11.1
Causes of disease		
Ischemic cerebral infarction	6 (42.9%)	6 (30%)
Hemorrhagic cerebral infarction	6 (42.9%)	8 (40%)
Traumatic brain injury	2 (14.2%)	6 (30%)

Values are mean ± standard deviation or the number of the participants with percentage in parentheses, where appropriate.

**Table 2:** Severity scores and Kozin’s classification scores.

	Total		High-Dose Group (*n* = 14)		Low-Dose Group (*n* = 20)	
	Before Treatment	After Treatment	Before Treatment	After Treatment	Before Treatment	After Treatment
Severity scores	4.86 ± 1.63	3.19 ± 1.68 *	4.69 ± 1.58	2.79 ± 1.74 *	4.98 ± 1.73	3.39 ± 1.78 *
Kozin’s classification scores	2.17 ± 0.68	1.61 ± 0.81 *	2.11 ± 0.72	1.39 ± 0.76 *	2.29 ± 0.79	1.73 ± 0.77 *

Values are mean ± standard deviation; * Statistical significance at *p* < 0.05.

**Table 3:** The radioisotope uptake ratio based on three-phase bone scintigraphy scans.

	Total		High-Dose Group (*n* = 14)		Low-Dose Group (*n* = 20)	
	Before Treatment	After Treatment	Before Treatment	After Treatment	Before Treatment	After Treatment
Blood pooling phase	1.44 ± 0.26	1.35 ± 0.27	1.43 ± 0.22	1.37 ± 0.38	1.43 ± 0.29	1.36 ± 0.27
Delayed phase	1.47 ± 0.43	1.41 ± 0.29	1.51 ± 0.39	1.37 ± 0.26	1.35 ± 0.24	1.42 ± 0.26

Values are mean ± standard deviation.

## Appendix A

**Table A1:** Severity score.

Score	Pain		Swelling of Hand
	Hand	Shoulder	
3: Severe	Touch	ER < 30°	Generalized edema, convexity
2: Moderate	ROM of MP joint	30° ≤ ER < 60°	Loss of skin crease
1: Mild	No hand pain	ER ≥ 60°	Loss of digit crease
0: No	No hand pain	No shoulder pain	No swelling

ROM.; Range of Motion; MP joints; Metacarpophalangeal joints; ER.; External rotation of shoulder.

## Appendix B

**Table A2:** Kozin’s classification.

Grade	
3 (definite)	Pain, edema, and vasomotor abnormalities all exist
2 (probable)	There is pain and there is one of edema or vasomotor abnormalities
1 (possible)	Edema and vasomotor abnormalities exist but pain is unclear
